# Coloration Characteristics of the Chinese Giant Salamander (*Andrias davidianus*) in Zhangjiajie and Their Associations with Water Quality

**DOI:** 10.3390/ani16172686

**Published:** 2026-08-28

**Authors:** Yuanfei Wang, Dafeng Li, Yifang Zhang, Qinghua Luo, Honghui Li, Jinping Wang, Zhuhu Yi, Chuanjin Li, Shouliang Luo, He Tian

**Affiliations:** 1 Hunan Engineering Technology Research Center for Amphibian and Reptile Resource Protection and Product Processing, College of Biological and Chemical Engineering, Changsha University, Changsha 410022, China; wangyf15615972515@163.com (Y.W.); ldf203540@163.com (D.L.); zyf33629@163.com (Y.Z.); lee19890925@163.com (H.L.); luosl2021@163.com (S.L.); tianhe970808@126.com (H.T.); 2 Zhangjiajie Livestock and Aquatic Products Affairs Center, Zhangjiajie 427000, China; 13407449136@163.com; 3 Institute of Agricultural Science and Technology Research in Zhangjiajie City, Zhangjiajie 427000, China; yizhuhu@163.com (Z.Y.); 18974480916@163.com (C.L.)

**Keywords:** markings, captive environment, association, conservation management

## Abstract

Body colour is an important phenotypic trait of the Chinese giant salamander and may reflect its responses to environmental variation. However, quantitative comparisons of body colour among captive environments remain limited. We examined 56 individuals from six breeding facilities in Zhangjiajie, China. The lightness, red–green coordinate and yellow–blue coordinate of the dorsal surface, ventral surface and body markings were measured, along with water quality variables at each facility. Analysis of variance, principal component analysis, cluster analysis and Pearson correlation analysis were used to compare populations, classify colour types and assess environmental relationships. Body-colour characteristics differed significantly among facilities, although overall colour differences between the body background and markings were not significant. Four colour types were identified, with 87.5% of individuals showing either an orange-brown dorsal surface with greyish-black markings or a grey dorsal surface with black markings. Body colour was significantly associated with water physicochemical variables. Higher pH and dissolved oxygen were mainly related to lower lightness and greater ventral redness, whereas higher total phosphorus and ammonia nitrogen were associated with increased lightness and yellowness. Water temperature mainly affected yellowness. These findings support captive-environment optimisation, body-colour management, conservation breeding and germplasm-resource protection.

## Simple Summary

Body colour is an important trait that can provide information about animal adaptation, population differences and conservation management. However, colour variation in captive Chinese giant salamanders and its potential links with rearing conditions remain poorly understood. In this study, we found substantial variation in body colour among captive populations, with individuals showing distinct colour patterns dominated by an orange-brown dorsal surface with greyish-black markings or a grey dorsal surface with black markings. Differences in aquatic environments were associated with variation in colour traits, suggesting that rearing conditions may contribute to the expression of body colour phenotypes. Our findings highlight the value of body colour assessment for understanding environmental associations with colour variation in captive Chinese giant salamanders and provide useful information for improving breeding practices and supporting conservation programmes.

## 1. Introduction

Colour is a highly functional trait that mediates interactions between organisms and their environment. It plays key roles in survival, performance, reproductive success and communication [1] and is frequently associated with other morphological, behavioural, physiological and life-history traits. Organismal colouration can be divided into two distinct components: body colour, which is determined by the visual properties of light reflected or scattered by pigments or nanostructures in specific body regions, including hue, brightness and saturation; and colour pattern, which refers to the spatial arrangement of patches that vary in colour, size, orientation, number and shape, such as spots, stripes or other markings [2]. Studying colour, including both its physical properties and spatial patterns, is therefore essential for understanding its roles in survival, performance, reproductive success and communication, as well as its evolution and diversification [3]. These two components of colouration can convey partly distinct biological information. The brightness and hue of body colour can independently influence predator learning and attack rates because predators may process chromatic and luminance cues separately from the spatial arrangement of colour patterns, allowing colour properties and pattern geometry to function as distinct components of warning signals [4]. Body colour may also play a role in sexual selection by signalling individual quality or social status [5], while darker colouration is associated with more effective thermoregulation [6] and greater protection against ultraviolet radiation. Colour patterns may function as warning signals that deter predators [7], increase the conspicuousness of aposematic prey at close range [8], and be associated with different escape behaviours [9]. They may also influence sexual selection [10], serve as indicators of individual quality or status, and facilitate interspecific recognition [11].

Quantitative studies of body colour have shown that its physical properties are closely associated with both ontogenetic stage [12] and environmental conditions, including temperature, substrate colour and water transparency [13,14,15]. Ontogenetic changes in colouration may help organisms cope with the multiple selective pressures acting on body colour in aquatic environments. Temperature-dependent colour changes have been observed in several amphibian species. For example, *Hyla crucifer* and *Hyla cinerea* exhibit darker body colouration at lower temperatures and lighter colouration under warmer conditions [12]. Although darker colouration at low temperatures may enhance thermoregulation, it may also reduce larval camouflage against light-coloured substrates. Substrate colour and water transparency can likewise influence colour expression in salamander larvae. For example, Arizona tiger salamander (*Ambystoma tigrinum nebulosum*) larvae generally exhibit darker colouration on black substrates and in clear water, but lighter colouration on white substrates and in turbid water [14].

Amphibians are well known for their remarkable diversity in body colour and colour patterns. As one of the largest extant amphibians, the Chinese giant salamander (*Andrias davidianus*) also exhibits considerable variation in body colouration. Previous studies have shown that adults are typically brown, yellowish-brown or greyish-brown, with irregular black or dark-brown markings, whereas juveniles are generally dark brown with black spots [16]. In addition to these common dark-coloured phenotypes, unusual colour variants have also been observed in captive-bred populations. Approximately 0.1% of captive-bred individuals have been reported to exhibit an albino phenotype, which is accompanied by delayed somatic growth and early mortality [17], while yellow phenotypes have also been documented. Molecular studies have further revealed that these colour variants are associated with differences in genes and regulatory pathways involved in melanogenesis. For example, transcriptomic analyses of albino individuals have shown reduced expression of genes involved in melanin synthesis [17], and other studies have identified pigmentation-related genes, including *TYR*, *TYRP1*, *ASIP*, *MITF* and *SLC24A5*, as being potentially involved in regulating skin pigmentation and body colour development in this species [18,19]. Moreover, recent research has shown that body colour in Chinese giant salamanders exhibits rhythmic changes across the light–dark cycle, becoming darker during light periods and lighter during dark periods. This suggests that body colour may be jointly regulated by external light conditions and endogenous physiological rhythms [16]. Collectively, these findings indicate that body colour diversity in Chinese giant salamanders may arise from the combined effects of genetic background, pigment-regulatory mechanisms and environmental responses.

Current research on body colour variation in Chinese giant salamanders has focused mainly on unusual colour phenotypes and their underlying molecular regulatory mechanisms, whereas quantitative studies comparing body colour characteristics across different captive-rearing environments and examining their associations with environmental factors remain limited. The Zhangjiajie Chinese giant salamander has relatively high muscle nutritional quality [20] and represents an economically and regionally important aquaculture resource. These characteristics highlight the importance of systematically characterising phenotypic variation within this recognised regional strain. Accordingly, this study investigated body colour variation in Chinese giant salamanders reared in Zhangjiajie and examined the relationships between body colour characteristics and water quality variables in captive facilities. The findings provide a scientific basis for optimising captive-rearing environments and improving body colour management. They also enhance our understanding of phenotypic plasticity and environmental adaptation under captive-breeding conditions, thereby supporting conservation breeding and germplasm-resource conservation in Chinese giant salamanders.

## 2. Materials and Methods

### 2.1. Sampling Sites

**Figure 1:**
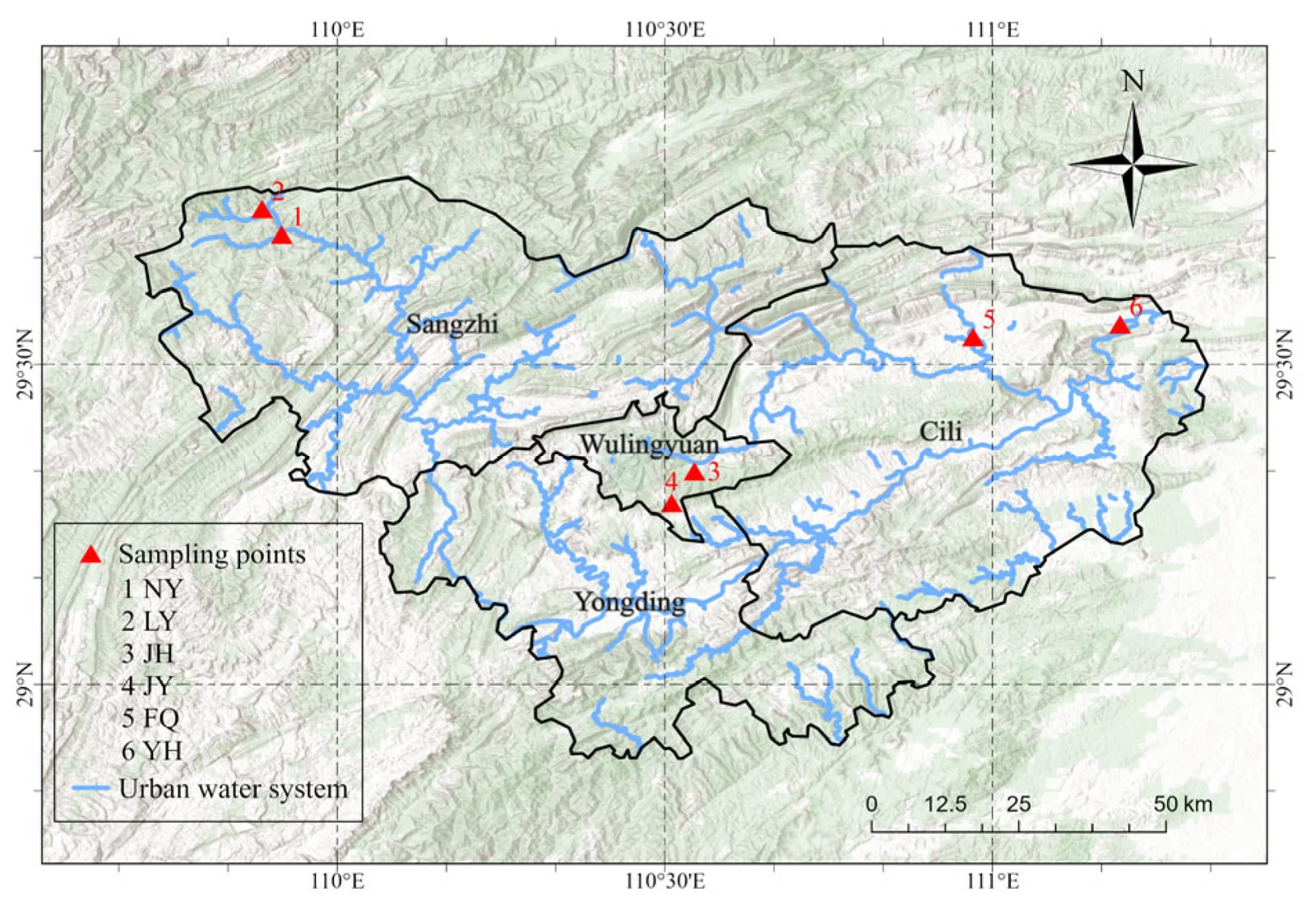
The sampling point distribution of Chinese giant salamanders in Zhangjiajie.

In August 2022, a total of 56 Chinese giant salamanders aged 2–5 years (body weight: 1.89 ± 0.71 kg; body length: 41.05 ± 4.87 cm) were obtained from six aquaculture farms in Zhangjiajie City, Hunan Province for body colour measurements (Figure 1; Table S1). All farms cultured the local Zhangjiajie strain at a rearing density of approximately 3–4 individuals per square metre. The salamanders were uniformly fed *Hemiculter leucisculus* once daily and had been maintained under these rearing conditions for at least six months before sampling.

### 2.2. Measured Variables

**Figure 2:**
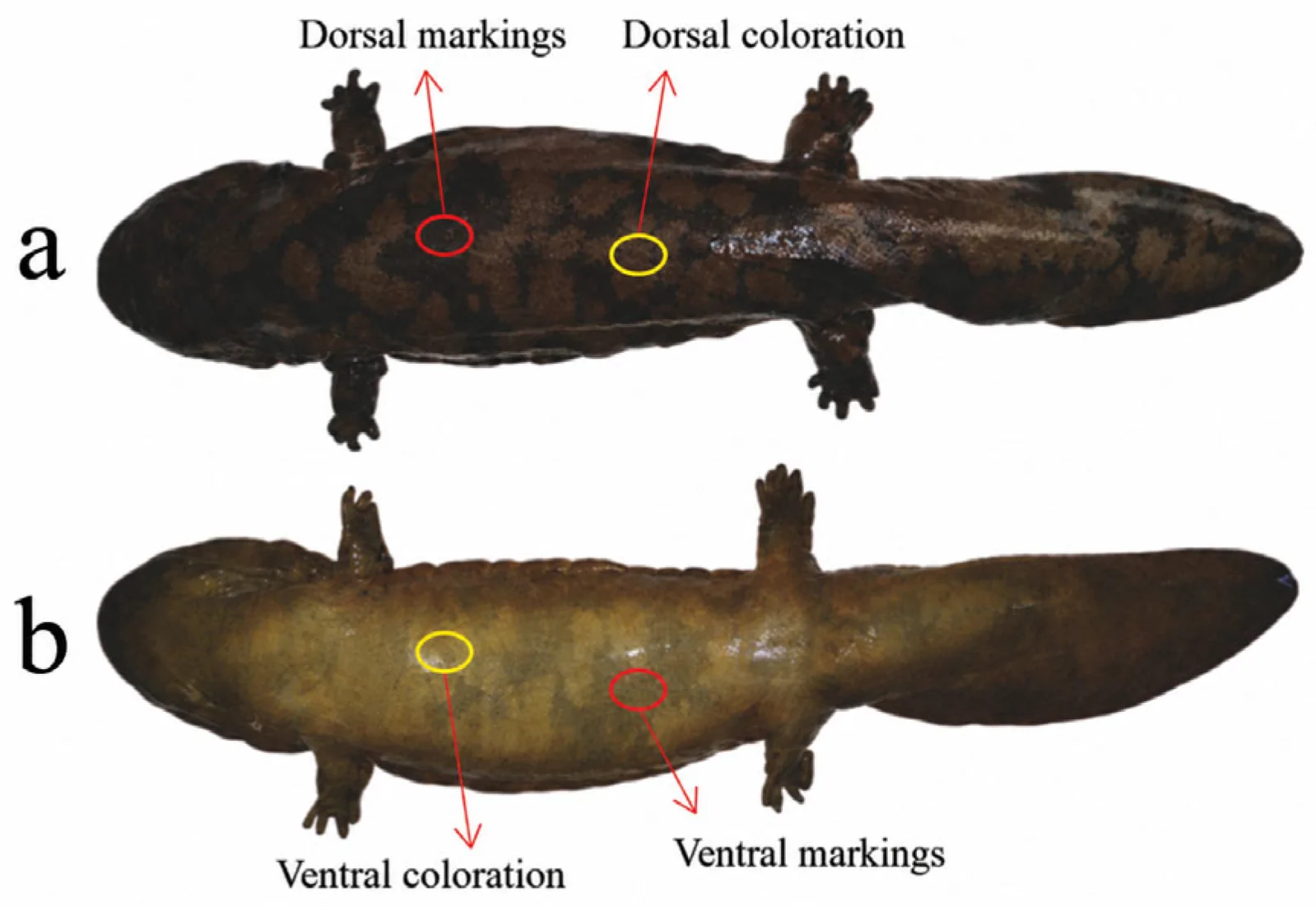
Measurement locations for body colour and markings in the Chinese giant salamander. (**a**) Dorsal body colour and markings; (**b**) ventral body colour and markings.

The distinction between body colour and markings was made by visual inspection based on the morphological classification criteria described by Fei (2006) [21]. Body colour was defined by the visual properties of a given body region, including hue, lightness and saturation, whereas colour pattern was defined by the spatial arrangement of patches differing in colour, size, orientation, number or shape. Accordingly, irregular patches on the surface of Chinese giant salamanders were classified as markings, whereas the surrounding coloured regions were classified as body colour (Figure 2). A colourimeter (NR20XE; 3nh Technology Co., Ltd., Shenzhen, China) was used to measure the colour parameters of the dorsal and ventral body colour and markings. Before measurement, the colourimeter was calibrated according to the manufacturer’s instructions. White calibration was performed using the supplied white calibration board, whereas black calibration was performed with the measuring aperture directed towards air in a dark environment free from nearby reflective objects. Colour measurements were conducted using the 45° optical geometry of the NR20XE colourimeter. Body colour was quantitatively assessed using the Commission Internationale de l’Éclairage L*a*b* (CIELAB) colour space, and the L*, a* and b* values were recorded. L* represents lightness and ranges from 0 to 100, with higher values indicating greater lightness; values of 0 and 100 correspond to black and reference white, respectively. The a* coordinate represents the green–red axis, where negative and positive a* values represent green and red components, respectively, with increasing values indicating a shift towards red and decreasing values indicating a shift towards green. The b* coordinate represents the blue–yellow axis, where negative and positive b* values represent blue and yellow components, respectively, with increasing values indicating a shift towards yellow and decreasing values indicating a shift towards blue [22].

At each breeding farm, water-quality variables were measured at three sampling points: the water inlet, rearing pond and water outlet. Water temperature (WT), dissolved oxygen (DO), pH, and electrical conductivity (EC) were measured using a portable multiparameter water quality meter (Hach HQ40d, Loveland, CO, USA) following previously described methods [23]. In accordance with the Environmental Quality Standard for Surface Water (GB 3838-2002) [24], chemical oxygen demand (COD) was determined using the dichromate method, with a detection limit of 2 mg L^−1^. Ammonia nitrogen (NH_3_-N) was determined using salicylate spectrophotometry, with a detection limit of 0.01 mg L^−1^. Total phosphorus (TP) was determined using ammonium molybdate spectrophotometry, with a detection limit of 0.01 mg L^−1^, and sulphide (S) was determined using methylene blue spectrophotometry, with a detection limit of 0.005 mg L^−1^. General hardness (GH) was determined by EDTA titration. For each breeding farm, the values measured at the three sampling points were averaged, and these farm-level mean values were used in subsequent correlation analyses with body colour characteristics.

### 2.3. Statistical Analysis

To quantify the colour difference between the body colour and adjacent markings at each measurement site within an individual Chinese giant salamander, the overall colour difference (ΔE) was calculated as follows:

(1) $$\Delta E\;=\;\sqrt{{\left(\Delta L^{*}\right)}^{2}+{\left(\Delta a^{*}\right)}^{2}+{\left(\Delta b^{*}\right)}^{2}}$$

where $\Delta L^{*}=L_{1}^{*}-L_{2}^{*}$, $\Delta a^{*}=a_{1}^{*}-a_{2}^{*}$, and $\Delta b^{*}=b_{1}^{*}-b_{2}^{*}$. $L_{1}^{*}$, $a_{1}^{*}$, and $b_{1}^{*}$ represent the body-colour parameters at a given measurement site, whereas $L_{2}^{*}$, $a_{2}^{*}$, and $b_{2}^{*}\;$ represent the colour parameters of the adjacent marking. A higher ΔE value indicates a greater colour difference between the body colour and the marking.

Statistical analyses were performed using SPSS 25.0 and Origin 2024. Normality and homogeneity of variances were assessed using the Shapiro–Wilk test and Levene’s test, respectively. Differences in body colour characteristics among breeding farms were assessed using one-way ANOVA, followed by Tukey’s post hoc test. Pearson correlation analysis was initially conducted among body colour variables, which were screened before principal component analysis (PCA) to reduce multicollinearity. For pairs of highly correlated variables (∣*r*∣ > 0.7), the variable with the higher mean absolute correlation with the remaining variables was excluded. The retained body colour variables were first subjected to PCA. Principal components with eigenvalues greater than 1 were retained according to the Kaiser criterion and were subsequently subjected to Varimax rotation. Based on the PCA loadings, eight representative body colour variables were selected for subsequent hierarchical cluster analysis. Hierarchical clustering was performed using squared Euclidean distance as the distance metric and average linkage between groups as the linkage method. Pearson correlation analysis was also used to examine the relationships between water quality variables and body colour characteristics. To account for multiple comparisons, *p* values from these correlation tests were adjusted using the Benjamini–Hochberg false discovery rate (FDR) procedure. Body colour data are presented as mean ± SD. Statistical significance was set at *p* < 0.05, with FDR-adjusted *p* values used for the water quality–body colour correlation analyses.

## 3. Results

### 3.1. Body Coloration Traits

**Figure 3:**
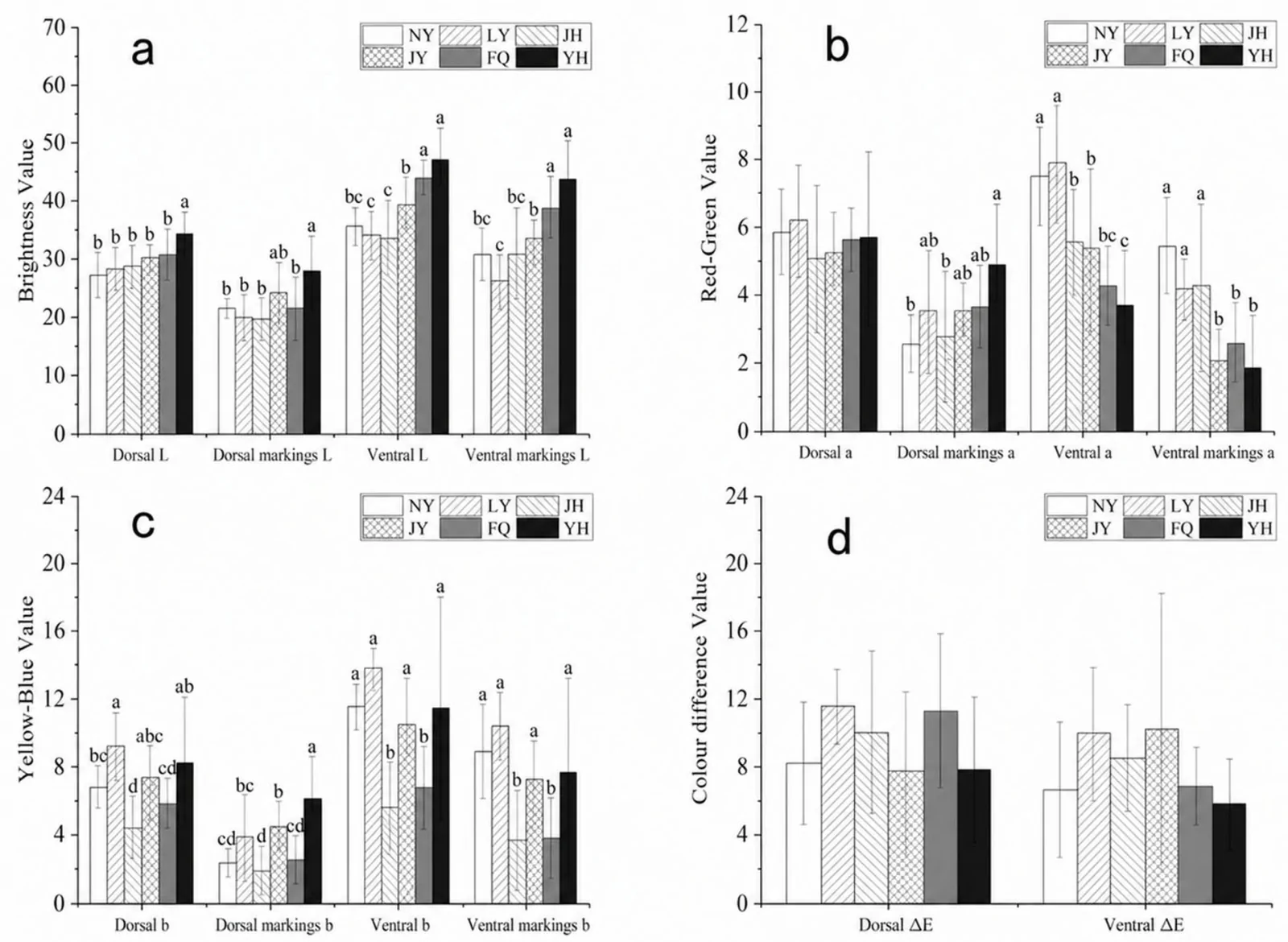
Variation in body colouration and markings of Chinese giant salamanders among breeding farms. (**a**) Brightness value; (**b**) red-green value; (**c**) yellow-blue value; (**d**) colour difference value. Different lowercase letters indicate significant differences among breeding farms for the same colour variable at *p* < 0.05, whereas values sharing the same letter are not significantly different.

Analysis of variance revealed significant differences among breeding-farm populations in the lightness (L*), red–green coordinate (a*) and yellow–blue coordinate (b*) of Chinese giant salamanders (Figure 3). The YH population exhibited the highest dorsal and ventral L* values, indicating the greatest overall lightness. The LY population showed the highest dorsal and ventral b* values and therefore the strongest yellow colouration. In contrast, the JH population had the lowest ventral b* value, indicating weaker yellow colouration. The YH population also had the highest a* value for dorsal markings but the lowest ventral a* value. However, ΔE did not differ significantly among populations.

**Figure 4:**
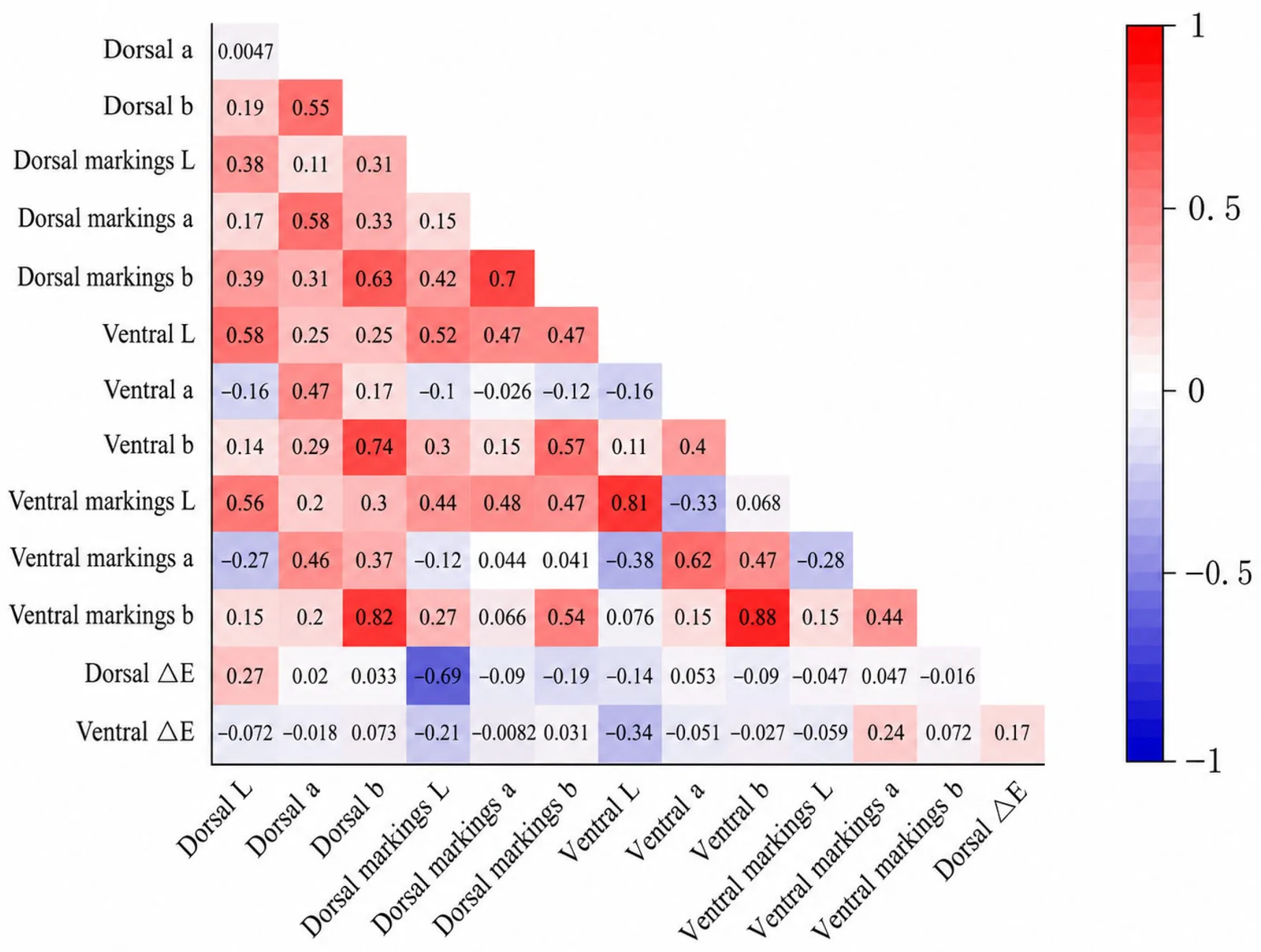
Pearson correlations among body colour and marking variables in Chinese giant salamanders.

Pearson correlation analysis showed that five pairs of body colour variables had absolute correlation coefficients greater than 0.7 (Figure 4): dorsal marking a* and dorsal marking b*, dorsal b* and ventral marking b*, dorsal b* and ventral b*, ventral marking L* and ventral L*, and ventral marking b* and ventral b*. After excluding dorsal b*, dorsal marking b*, ventral marking L* and ventral marking b*, 10 body colour variables were retained for principal component analysis.

Based on the Kaiser criterion (eigenvalues > 1), four principal components were retained, collectively explaining 75.625% of the total variance. Varimax rotation was applied to these four components, yielding the rotated component matrix (Table 1). The variables with high loadings on principal components 1 and 2 included dorsal L*, ventral L*, dorsal a* and dorsal marking a*, and these components were interpreted as primarily representing dorsal body colouration. Dorsal marking L* and dorsal ΔE loaded strongly on principal component 3, which mainly represented dorsal colour difference. Ventral a* and ventral b* showed high loadings on principal component 4, which primarily represented ventral body colouration. Accordingly, eight body colour variables—dorsal L*, dorsal a*, dorsal marking L*, dorsal marking a*, ventral L*, ventral a*, ventral b* and dorsal ΔE—were selected as representative variables for subsequent body colour clustering.

### 3.2. Cluster Analysis of Body Colour

**Figure 5:**
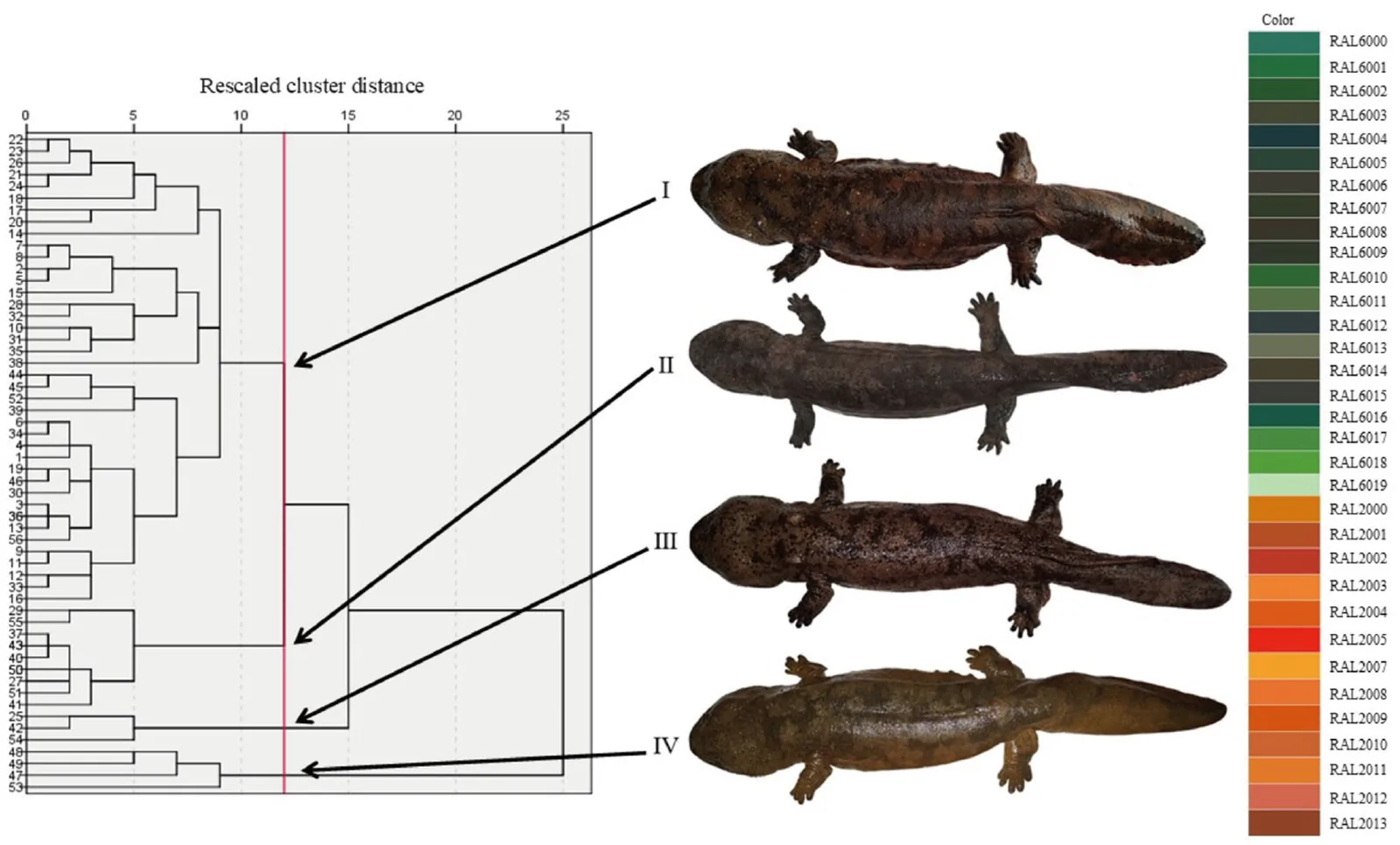
Hierarchical clustering dendrogram of Chinese giant salamanders based on body colour variables.

**Figure 6:**
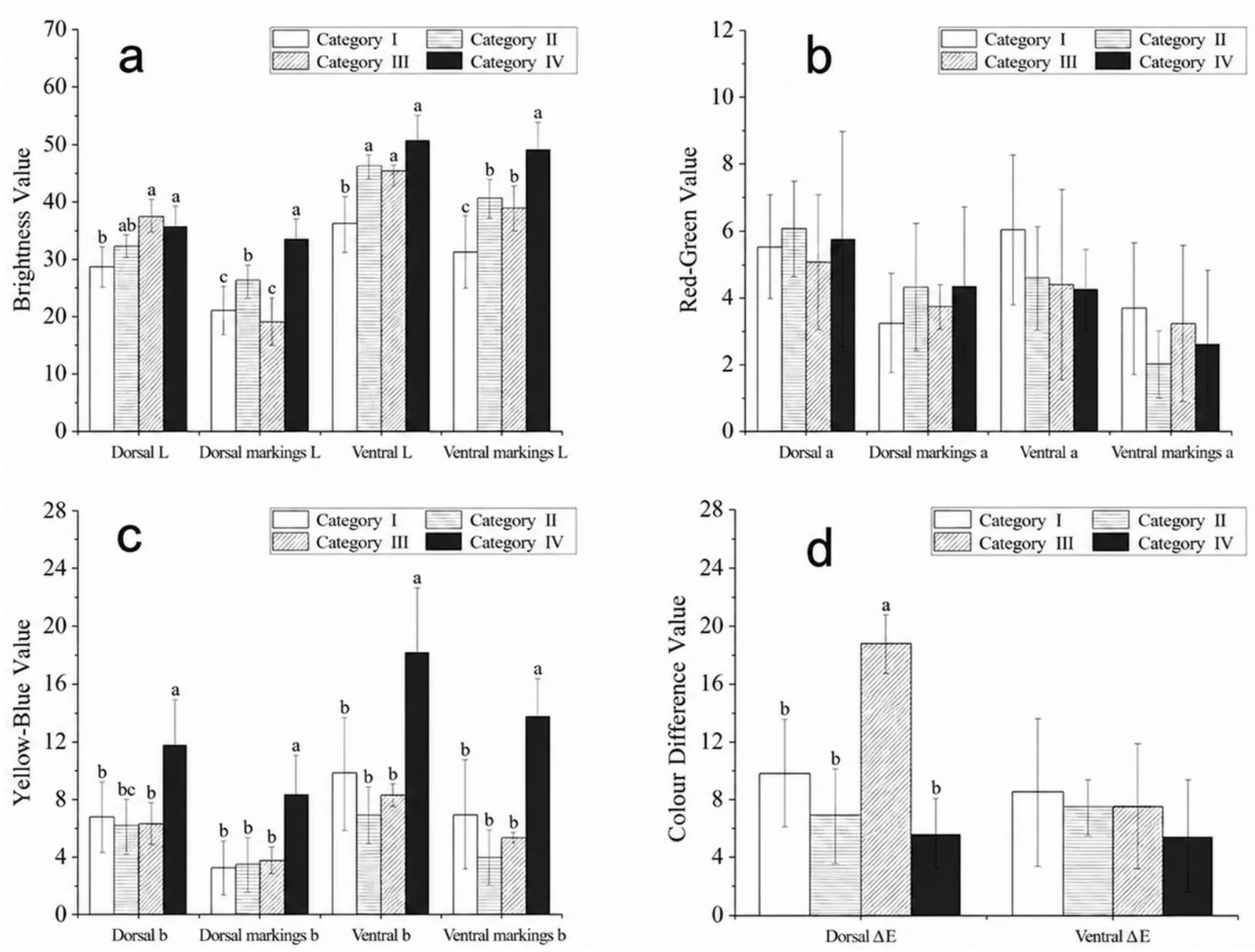
Differences in body coloration among categories of Chinese giant salamanders. (**a**) Brightness value; (**b**) red-green value; (**c**) yellow-blue value; (**d**) colour difference value. Different lowercase letters indicate significant differences among categories for the same colour variable at *p* < 0.05, whereas values sharing the same letter are not significantly different.

Hierarchical cluster analysis classified the 56 Chinese giant salamanders into four clusters, comprising 40 individuals in Cluster I, nine in Cluster II, three in Cluster III and four in Cluster IV (Figure 5). The dendrogram showed clear separation among the four clusters, with Cluster IV being the most distinct from the remaining clusters, indicating that its overall body colour characteristics differed most strongly from those of the other clusters. Analysis of variance revealed significant differences in multiple body colour variables among the four clusters (Figure 6). Cluster IV exhibited significantly greater lightness and yellowness than the other clusters, whereas Cluster III showed the greatest dorsal colour difference. No significant differences in the red–green coordinate were detected among clusters. Specifically, individuals in Cluster I had orange-brown dorsal colouration with greyish-black markings; those in Cluster II had grey dorsal colouration with black markings; those in Cluster III had dark-brown dorsal colouration with black markings; and those in Cluster IV had yellowish-brown dorsal colouration with greyish-black markings (Figure 5).

### 3.3. Relationship Between Water Quality and Body Coloration

One-way ANOVA revealed significant differences among breeding farms in WT, DO, EC and GH (*p* < 0.05). The YH farm had the highest WT but the lowest DO concentration, reflecting a distinct combination of high temperature and low oxygen availability. In contrast, the JH farm had the lowest WT, together with relatively high DO concentrations and pH values. EC was relatively high at the JH, YH and FQ farms and relatively low at the LY and JY farms. GH was highest at the YH farm and lowest at the JY farm. No significant differences among farms were detected in COD, TP, NH_3_-N or S (*p* > 0.05) (Table 2).

**Figure 7:**
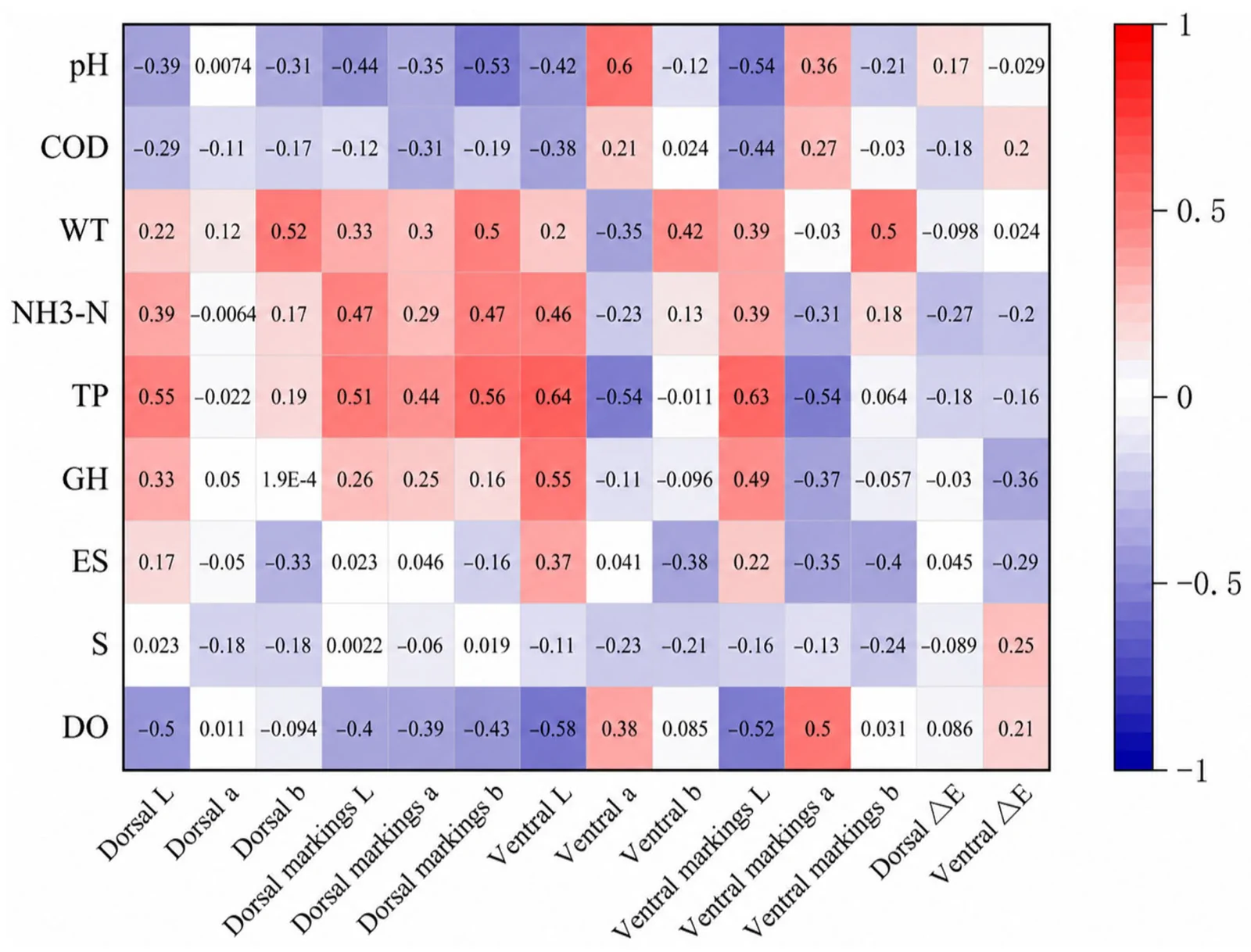
Pearson correlations between water quality variables and body colour characteristics in Chinese giant salamanders.

Pearson correlation analysis between water quality variables and body colour characteristics showed that both pH and DO were significantly negatively correlated with dorsal L*, dorsal marking L*, dorsal marking a*, dorsal marking b* and ventral L*, but significantly positively correlated with ventral marking a* and ventral a* (all *p* < 0.05). WT was significantly positively correlated with dorsal b* (*r* = 0.52, *p* < 0.05), dorsal marking b* (*r* = 0.5, *p* < 0.05), ventral b* (*r* = 0.42, *p* < 0.05) and ventral marking b* (*r* = 0.5, *p* < 0.05), and significantly negatively correlated with ventral a* (*r* = −0.35, *p* < 0.05). TP was significantly positively correlated with dorsal L* (*r* = 0.55, *p* < 0.05), dorsal marking L* (*r* = 0.51, *p* < 0.05), ventral L* (*r* = 0.64, *p* < 0.05) and ventral marking L* (*r* = 0.63, *p* < 0.05), but significantly negatively correlated with ventral marking a* (*r* = −0.54, *p* < 0.05) and ventral a* (*r* = −0.54, *p* < 0.05). NH_3_-N was significantly positively correlated with dorsal L* (*r* = 0.39, *p* < 0.05), dorsal marking L* (*r* = 0.47, *p* < 0.05), dorsal marking b* (*r* = 0.47, *p* < 0.05) and ventral L* (*r* = 0.46, *p* < 0.05), and significantly negatively correlated with dorsal ΔE (*r* = −0.27, *p* < 0.05) (Figure 7).

## 4. Discussion

Body colour is an important morphological trait with considerable value for species identification and the differentiation of geographical populations. Analysis of body colour in captive Chinese giant salamanders from Zhangjiajie revealed significant differences among breeding farms. Twelve parameters, including dorsal lightness, the dorsal red–green coordinate and dorsal marking lightness, were useful for characterising body colour variation, with dorsal colouration and markings providing particularly important discriminatory information. Chinese giant salamanders from Zhangjiajie exhibited four body colour types: reddish-brown dorsal colouration, similar to that of Chinese giant salamanders from Longli, Guizhou [25]; yellowish-brown dorsal colouration, similar to that of individuals from Sangzhi, Hunan; dark-brown dorsal colouration, similar to that of individuals from Jiangkou, Guizhou [26]; and greyish-black dorsal colouration, often accompanied by black block-like or mottled markings. This pattern is consistent with the pronounced body colour variation reported among geographical populations of other amphibians. Barzaghi et al. (2022) found colour differences among geographically proximate populations of the fire salamander (*Salamandra salamandra*) [27], whereas Blackburn (2021) showed that geographical populations of African long-fingered frogs (*Arthroleptidae Cardioglossa*) could be distinguished by body colour [28]. The body colour differences observed in the present study may therefore provide useful morphological information for distinguishing Chinese giant salamanders among breeding-farm populations. Chinese giant salamanders from the six breeding farms also differed markedly in individual colour parameters. Individuals from the YH population had the highest dorsal, dorsal marking and ventral lightness values and therefore the lightest overall colouration. Their dorsal markings also showed stronger red and yellow colouration, whereas their ventral markings and ventral surfaces showed weaker red colouration. Individuals from the LY population exhibited the strongest overall yellow colouration, with yellower dorsal surfaces, ventral markings and ventral surfaces, as well as the strongest red colouration on the ventral surface. Individuals from the JH and FQ populations showed weaker yellow colouration, with their ventral markings and ventral surfaces tending towards blue. Individuals from the FQ population also exhibited greater ventral marking lightness. Individuals from the NY population had redder ventral markings and ventral surfaces but intermediate lightness and yellow colouration. These results indicate multidimensional differentiation in body colour among Chinese giant salamanders from different breeding farms, consistent with the pronounced variation in colour patterns observed in European fire salamanders (*Salamandra salamandra*) across different environmental conditions [27]. In amphibians, skin colour is generated through the combined effects of different chromatophore types, including melanophores, xanthophores, and iridophores, and changes in their abundance, distribution, or physiological state can alter both body lightness and chromatic components. Experimental studies have shown that amphibian pigmentation can respond plastically to environmental conditions through changes in melanophore- and xanthophore-related processes [29]. Environmental differences among breeding farms, including variation in water quality, may therefore contribute to the contrasting colour characteristics observed among these groups.

Intra- and interspecific variation in animal body colouration results from the combined effects of multiple factors over the course of long-term evolution, including physiological constraints, sexual selection pressures and natural selection [1,30]. Body colouration is not only an important trait for species recognition but also an important manifestation of adaptation to environmental conditions, with critical consequences for individual survival, growth and reproductive success [31]. Recent studies have demonstrated that environmental factors play an important role in shaping animal colouration. Barzaghi et al. (2022) found that environmental conditions and food resource availability had stronger effects on dorsal colour patterns in salamanders than local adaptation [27]. Water pollution levels are significantly associated with body colour characteristics in frogs of the genus *Pelophylax*, with colour variation closely linked to eutrophication and increased loads of organic pollutants in aquatic environments [32]. Our results showed that water temperature was positively correlated with yellow colouration in Chinese giant salamanders, as indicated by positive associations with dorsal b* (*r* = 0.52, *p* < 0.01), dorsal marking b* (*r* = 0.50, *p* < 0.01), ventral b* (*r* = 0.42, *p* < 0.01) and ventral marking b* (*r* = 0.50, *p* < 0.01), suggesting that higher temperatures may be associated with enhanced yellow colouration. This finding is consistent with the general pattern in which temperature influences colour variation in ectothermic animals [33]. For example, low temperatures can induce frogs of the genus *Rhacophorus* to rapidly develop darker colouration, thereby enhancing heat absorption, whereas individuals under high-temperature conditions tend to maintain lighter colouration to reduce heat gain [33]. King et al. (1994) also observed lighter body colouration in the green treefrog (*Hyla cinerea*) at higher temperatures [15]. Malik et al. (2023) [34] elucidated the molecular mechanisms underlying temperature-induced colour responses in Chinese giant salamanders. Under low-temperature conditions, TRPM8-mediated melanophore migration and dispersion lead to darker colouration, thereby enhancing heat absorption; under higher-temperature conditions, increased accumulation of carotenoids and other yellow pigments enhances yellow colouration. These contrasting responses may arise from differences in how melanogenic and carotenoid-based pigmentation systems respond to temperature variation [15,35], reflecting the complex regulatory strategies evolved by amphibians to cope with fluctuations in environmental temperature.

Our study showed that pH and DO were significantly associated with multiple body colour characteristics in Chinese giant salamanders. Specifically, pH was significantly negatively correlated with dorsal L*, dorsal marking a* and dorsal marking b*, but significantly positively correlated with ventral marking a*. This pattern was partly consistent with observations in tadpoles of the Asian black-spined toad (*Duttaphrynus melanostictus*) [36], although differences among studies may be related to variation in pigment composition, developmental stage and environmental context. DO was negatively correlated with dorsal L*, dorsal marking L*, ventral L* and ventral marking L*, but positively correlated with ventral a* and ventral marking a*, indicating that higher DO levels were generally associated with lower body surface lightness and stronger red colouration on the ventral surface. This pattern differed from that reported by Abdel (2019) in fish, suggesting that body colouration may respond differently to changes in aquatic conditions among taxonomic groups [37]. The associations of pH and DO with body colouration may involve multiple physiological and chemical processes. For example, alkaline conditions may alter the chemical forms of metal ions in water, such as Fe^3+^ and Cu^2+^, and the stability of their binding to pigments [38]. Low DO conditions may also influence melanin synthesis through hypoxia-related signalling pathways [39]. However, the present study did not directly measure bile pigments, melanophore activity, metal-ion speciation or hypoxia-inducible factors; therefore, these potential mechanisms require verification through further physiological and molecular experiments. Our study also showed that TP concentration in the water was positively associated with body colour lightness in Chinese giant salamanders. Phosphorus status has been linked to growth, antioxidant capacity, skin structural integrity and the regulation of signalling pathways such as NF-κB, TOR and Nrf2 in aquatic animals [40,41], suggesting a possible physiological link between phosphorus availability and colour expression. However, TP concentration in aquaculture water is also strongly influenced by husbandry conditions and nutrient inputs. Differences among farms in feed inputs, water sources and stocking density may therefore affect TP concentrations while simultaneously influencing animal growth, physiological condition and body colour. Thus, the observed TP–colour relationship may reflect, at least in part, broader differences in rearing conditions rather than a direct effect of TP itself. This association highlights the need for controlled experiments that manipulate phosphorus availability while standardising other husbandry factors to determine whether TP has an independent effect on body colour lightness. Overall, aquatic environmental conditions differed significantly among breeding farms, particularly in key variables such as WT and DO. These differences may be associated with variation in growth and development, body colour phenotypes, and physiological condition in Chinese giant salamanders. Higher WT was associated with greater yellow colouration, suggesting that avoiding pronounced decreases in WT and maintaining relatively stable thermal conditions may help preserve yellow colour expression. Higher pH was generally associated with lower body colour lightness, indicating that unnecessary increases in pH should be avoided when lighter body colour phenotypes are desired. Although higher DO was also associated with lower lightness, DO should be maintained at adequate and stable levels to safeguard animal health rather than manipulated specifically to alter colour. Together, these results support routine monitoring and stabilisation of WT, DO and pH as practical components of captive-rearing management aimed at maintaining consistent body colour phenotypes.

## 5. Conclusions

Body colour variation in Chinese giant salamanders differed among breeding farms and was closely associated with aquatic environmental conditions, highlighting its potential value for phenotypic assessment and water quality management. However, several limitations should be considered. This study was based on 56 individuals from six breeding farms sampled during a single period, and the observed relationships between water quality and body colour were therefore correlational rather than causal. In addition, age and sex were not specifically balanced among farms or among the colour clusters, which may have contributed to the observed body colour differences. Future studies should incorporate controlled experiments and repeated measurements across longer time periods to distinguish temporal variation from the effects of individual environmental factors.

## Figures and Tables

**Table 1:** The principal component analysis of body colour and marking variables.

Variables	Component			
	1	2	3	4
Dorsal L*	0.839 *	0.023	0.098	0.114
Dorsal a*	−0.060	0.893 *	−0.020	0.260
Dorsal marking L*	0.426	0.020	−0.824 *	0.235
Dorsal marking a*	0.322	0.811 *	−0.083	−0.117
Ventral marking a*	−0.523	0.409	0.058	0.452
Ventral L*	0.823 *	0.317	−0.160	−0.050
Ventral a*	−0.445	0.328	0.128	0.728 *
Ventral b*	0.228	0.006	−0.096	0.842 *
Dorsal ΔE	0.145	0.027	0.934 *	0.002
Ventral ΔE	0.023	−0.123	0.430	0.318
Eigenvalue	2.516	2.308	1.572	1.167
Variance explained (%)	25.160	23.079	15.720	11.667
Cumulative variance explained (%)	25.160	48.238	63.958	75.625

Note: * indicates an absolute component loading greater than 0.700. Dorsal b*, Dorsal marking b*, Ventral marking L*, and Ventral marking b* were excluded to reduce multicollinearity.

**Table 2:** Water quality among breeding farms.

Farm	NY	LY	JH	JY	FQ	YH	Mean ± SD
pH	7.98 ± 0.05 b	7.78 ± 0.07 bc	8.31 ± 0.25 a	7.27 ± 0.31 e	7.64 ± 0.06 cd	7.40 ± 0.04 de	7.73 ± 0.38
WT (°C)	20.73 ± 0.31 b	22.63 ± 0.71 a	16.70 ± 1.73 c	22.87 ± 0.42 a	21.03 ± 0.35 b	26.27 ± 0.31 a	21.11 ± 2.34
DO (mg/L)	9.81 ± 0.50 a	8.90 ± 0.02 b	7.73 ± 0.81 c	9.12 ± 0.11 ab	8.11 ± 0.16 c	4.34 ± 0.19 d	8.00 ± 1.94
EC (s/m)	380.33 ± 4.93 d	193.73 ± 2.38 e	532.33 ± 13.2 a	137.70 ± 2.55 f	479.67 ± 13.28 c	503 ± 3.61 b	371.13 ± 168.05
COD (mg/L)	8.28 ± 1.06	4.15 ± 0.00	7.53 ± 6.39	9.78 ± 1.06	3.01 ± 0.00	2.93 ± 0.10	5.95 ± 2.95
TP (mg/L)	0.01 ± 0.00	0.02 ± 0.00	0.03 ± 0.01	0.05 ± 0.00	0.04 ± 0.00	0.11 ± 0.10	0.04 ± 0.04
NH_3_-N (mg/L)	1.81 ± 0.00	0.11 ± 0.00	0.16 ± 0.08	0.16 ± 0.19	0.10 ± 0.05	0.31 ± 0.28	0.17 ± 0.08
S (mg/L)	-	0.01 ± 0.00	0.03 ± 0.00	0.04 ± 0.00	0.01 ± 0.00	0.01 ± 0.00	0.02 ± 0.01
GH (mg/L)	186.85 ± 7.14 c	95.95 ± 7.14 e	146.45 ± 7.14 d	40.40 ± 14.28 f	212.1 ± 14.28 b	297.95 ± 7.14 a	163.28 ± 90.54

Note: “-” indicates that the sulphide concentration was below 0.01 mg/L. Within the same row, means sharing the same letter are not significantly different according to Tukey’s post hoc test (*p* > 0.05).

## Data Availability

The original contributions presented in this study are included in the article/Supplementary Material. Further inquiries can be directed to the corresponding author.

## Supplementary Materials

The following supporting information can be downloaded at https://www.mdpi.com/article/10.3390/ani16172686/s1, Table S1: Sampling sites of Chinese giant salamanders in Zhangjiajie.

## Abbreviations

The following abbreviations are used in this manuscript:

NY	Niyuan
LY	Liyuan
JH	Jiuhu
JY	Jiangyong
FQ	Fuquan
YH	Yuanhui
WT	Water temperature
DO	Dissolved oxygen
EC	Electrical conductivity
COD	Chemical oxygen demand
NH_3_-N	Ammonia nitrogen
TP	Total phosphorus
S	Sulphide
GH	General hardness
