# Supplementary material for: Coloration Characteristics of the Chinese Giant Salamander (Andrias davidianus) in Zhangjiajie and Their Associations with Water Quality
Source: Animals (Basel). 2026 Aug 28;16(17):2686. doi: 10.3390/ani16172686 (PMC13564272; doi:10.3390/ani16172686)
Supplement: Supplementary file 1 [file animals-16-02686-s001.zip › animals-4492506-supplementary.pdf]

Supporting information for online publication

Coloration characteristics of the Chinese giant salamander (*Andrias davidianus*) in Zhangjiajie and their associations with water quality

Table S1. Sampling sites of Chinese giant salamanders in Zhangjiajie.

Number	Sampling sites	longitude and latitude	Altitude	Administrative zone	Number of samples
1	Sangzhi County Wudaoshui Niyuan Breeding Cooperative (NY)	29.70°N, 109.91°E	415 m	Wudaoshui Town, Sangzhi County	8
2	Zhangjiajie Liyuan Biotechnology Co., Ltd. (LY)	29.74°N, 109.88°E	452 m	Wudaoshui Town, Sangzhi County	8
3	Zhangjiajie Giant Salamander Jiuhu Center (JH)	29.33°N, 110.54°E	351 m	Wulingyuan District Junliping Office	10
4	Zhangjiajie Jiangyong Giant Salamander Breeding Cooperative (JY)	29.28°N, 110.51°E	355 m	Xiehe Township, Wulingyuan District	10
5	Cili County Fuquan Giant Salamander Breeding Farm (FQ)	29.54°N, 110.97°E	136 m	Yangliupu Township, Cili County	10
6	Zhangjiajie Yuanhui Giant Salamander Breeding Professional Cooperative (YH)	29.56°N, 111.19°E	154 m	Tongjinpu Town, Cili County	10
